# Efficacy of pramipexole on quality of life in patients with Parkinson’s disease: a systematic review and meta-analysis

**DOI:** 10.1186/s12883-022-02830-y

**Published:** 2022-08-25

**Authors:** Tao Li, Shuang Zou, Zijuan Zhang, Meiruo Liu, Zhanhua Liang

**Affiliations:** 1 Department of Neurology, First Affiliated Hospital of Dalian Medical University, Dalian, People’s Republic of China; 2 Present Address: Information Centre, The Second Affiliated Hospital of Dalian Medical University, Dalian, People’s Republic of China; 3 Boehringer Ingelheim (China) Investment Co. Ltd, Shanghai, People’s Republic of China; 4 Present Address: Medical Affairs, Biogen Biotechnology (Shanghai) Co. Ltd, Shanghai, People’s Republic of China

**Keywords:** Parkinson’s disease, Pramipexole, Meta-analysis, PDQ-39

## Abstract

**Background:**

Quality of life (QoL) in patients with Parkinson’s disease (PD) is increasingly used as an efficacy outcome in clinical studies of PD to evaluate the impact of treatment from the patient’s perspective. Studies demonstrating the treatment effect of pramipexole on QoL remain inconclusive. This study aims to evaluate the effect of pramipexole on QoL in patients with PD by conducting a systematic review and meta-analysis of existing clinical trials.

**Methods:**

A systematic literature search of PubMed, Embase and the Cochrane Library was performed from inception to 30 April 2022 to identify randomised, placebo-controlled trials of patients with idiopathic PD receiving pramipexole, who reported a change from baseline in their QoL as measured by the 39-item Parkinson’s Disease Questionnaire (PDQ-39). Risk of bias was independently assessed by two reviewers using the Cochrane Collaboration’s tool for bias assessment.

**Results:**

Of 80 eligible articles screened, six trials consisting of at least 2000 patients with early or advanced PD were included. From the synthesis of all six selected trials, a significant mean change from baseline in the PDQ-39 total score of –2.49 (95% CI, –3.43 to –1.54; *p* < 0.0001) was observed with pramipexole compared with placebo. A trend toward improvement in QoL was consistently observed among patients who received optimal doses of pramipexole (≥ 80% of the study population on 1.5 mg dosage), regardless of disease severity (advanced versus early) or baseline QoL levels.

**Conclusion:**

This meta-analysis provides evidence for the potential treatment benefit of pramipexole in improving QoL in patients with PD.

**Supplementary Information:**

The online version contains supplementary material available at 10.1186/s12883-022-02830-y.

## Plain Language Summary

Parkinson’s disease is a chronic, progressive nervous system disorder with no known cure. Patients may experience a number of symptoms including stiffness, slowness, and uncontrollable muscle movements, all of which impact their quality of life. Most clinical studies in Parkinson’s disease measure the effect of treatment on improving symptoms, but other aspects such as quality of life are often overlooked. It is important to include quality of life measures in clinical studies of Parkinson’s disease, such as the 39-item Parkinson’s disease questionnaire (PDQ-39), to understand the impact of treatment from patients’ perspectives. Pramipexole is a dopamine agonist that is well-tolerated and effective at treating Parkinson’s disease symptoms. However, studies examining its effect on quality of life are inconclusive. This meta-analysis of existing clinical trials therefore aimed to evaluate the effect of pramipexole on quality of life in patients with Parkinson’s disease. Six trials consisting of at least 2000 patients with early or advanced Parkinson’s disease receiving treatment with pramipexole were included in this meta-analysis. Analysis of these six trials found a significant improvement in PDQ-39 total score with pramipexole compared with placebo. This meta-analysis provides new evidence for the potential treatment benefit of pramipexole in improving quality of life in patients with Parkinson’s disease.

**Supplementary Information:**

The online version contains supplementary material available at 10.1186/s12883-022-02830-y.

## Background

Parkinson’s disease (PD) is a chronic, progressive disease that has no curative therapy and it is the second most common neurodegenerative disease worldwide [1]. The disease is typically characterised by motor symptoms, including difficulty with movement, slowness, freezing, dyskinesia and fluctuations, as well as the increasing recognition of non-motor symptoms [2], all of which play an important role in patients’ quality of life (QoL) [3, 4]. As a multidimensional concept, QoL broadly reflects the impact of the disease and treatment from the patient’s perspective through self-reported measures and is increasingly recognised as an important outcome in the management of PD [5–7].

Pharmacological treatments such as dopamine agonists have been shown to improve motor symptoms of PD and also improvements in patients’ QoL [8, 9]. Pramipexole is a non-ergolinic, D3-preferring dopamine agonist that is generally well tolerated and efficacious in treating motor symptoms in both early and advanced PD, available as both immediate or extended release formulations with similar clinical efficacy and safety profiles [10, 11]. Pramipexole, as initial monotherapy or adjunctive therapy to levodopa, is efficacious in PD in preventing or delaying motor fluctuations or dyskinesia associated with long-term use of levodopa [8]. Prior literature reviews [5, 12] and a systematic review [9] have attempted to investigate the effect of dopamine agonists (including pramipexole) on QoL in patients with PD. These findings remain inconclusive due to the high variability in study designs and heterogeneous outcome measures of QoL applied in the selected trials [9]. Of the many generic measures of QoL, few are PD-specific. The 39-item Parkinson's Disease Questionnaire (PDQ-39) is the most widely used PD-specific health-related QoL questionnaire [13] and has demonstrated good reliability, validity, responsiveness and reproducibility [14, 15]. The present study aimed to investigate whether pramipexole is more efficacious than placebo in improving QoL in patients with PD, as measured by PDQ-39, through an updated systematic review and meta-analysis of existing trials. Additionally, we explored whether these benefits are consistent in patients with different PD characteristics.

## Materials and methods

### Eligibility criteria

For this meta-analysis, we included studies that met all of the following criteria: (i) randomised, placebo-controlled trial; (ii) comprised patients who were diagnosed with idiopathic PD in any stage, regardless of age, sex, location and race; (iii) comprised patients who were receiving pramipexole, alone or in combination with other anti-Parkinsonian treatments as an intervention; (iv) included the assessment of change in QoL from baseline as measured by PDQ-39. Studies were excluded if they were not published in English or consisted of a specific clinical population, such as those with existing comorbidities.

### Literature search

A computerised systematic search for all eligible studies from inception up to 30 April 2022 was conducted using PubMed, Embase and the Cochrane Library. The search strategy was adapted according to database and included combinations of the following terms: (Parkinson OR Parkinson’s disease) and (pramipexole OR sifrol OR Mirapex) and (randomized controlled trial OR rct OR placebo OR drug therapy OR random* OR trial OR controlled OR group OR trials OR groups).

The review format adhered to the updated Preferred Reporting Items for Systematic Reviews and Meta-Analyses (PRISMA) statement [16].

### Study selection and data extraction

Two investigators (ZZ and SZ) independently retrieved data and assessed study eligibility according to titles and/or abstracts. Relevant data (general information, study design, patient characteristics, treatment details, mean differences in PDQ-39 total score from baseline to last available follow-up) were extracted (primarily by ML) into an Excel database and independently examined by a second reviewer (ZZ). The estimate was the mean difference between the pramipexole versus placebo group, and a corresponding standard error (SE) was extracted or derived from confidence intervals (CIs) if the SE was not available from the source publication. For studies where mean differences were not available, the data were requested from the study authors, public sources or owners of the study database; these requests were documented.

### Risk of bias assessment

Two reviewers (ZZ and ML) independently evaluated the selected studies using the Cochrane Collaboration’s tool for assessing risk of bias (RoB 2 tool) in randomised trials [17, 18]. The quality of each study was evaluated for methodological bias in selection, performance, detection, attrition and reporting, as well as for other potential sources of bias. Inter-rater disagreement was minimal and resolved through discussion and re-examination of each particular study in question with the input of a third reviewer (ZL).

### Statistical analysis

To examine differences between treatments, we compared the mean change in the PDQ-39 total score between treatment groups from baseline to last available follow-up. The means were pooled where multiple pramipexole dose groups or formulations (immediate/extended release) were tested in a single study. The common effect was calculated using the inverse variance method, which derives a weighted average of treatment effects estimated from individual studies. The weights were chosen to be the reciprocal of the variance of the effect estimate from a single study, reflecting the amount of information from individual studies [17]. The Q and I^2^ statistics were used to assess heterogeneity for the variation in true-effect sizes across the included studies; a *p*-value below 0.10 for the Q statistic and/or I^2^ index above 50% indicated significant heterogeneity. If heterogeneity was detected, a random-effects model was applied as a supplementary analysis to compare the pooled means; otherwise, a fixed-effects model was applied, which used the inverse variance method to estimate the pooled mean difference between the pramipexole and placebo groups for all eligible trials, along with 95% CIs and two-sided *p*-values. Because of the few studies (< 10) identified within the scope of this analysis, funnel plot assessment for potential publication bias was not undertaken as the statistical power would be too low to determine asymmetry [19].

For continuous data, pooled means, 95% CIs and two-sided *p*-values were calculated using a fixed-effects model to estimate mean differences between the pramipexole and placebo treatment groups for all eligible trials. Sensitivity analyses explored whether the outcome of interest was influenced by studies with risk of bias or non-normal distribution.

Additional exploratory subgroup meta-analyses were conducted after grouping selected studies according to: (i) pramipexole treatment dose: studies with ≥ 80% of patients receiving the recommended daily dose of ≥ 1.5 mg were categorised as “optimal dose” groups [20, 21] and those with a daily dose < 1.5 mg were considered “low dose” groups; (ii) baseline disease stage: advanced or early PD, according to the disease severity of the respective original studies; (iii) baseline QoL level: the studies were grouped into high (≥ 25) or low (< 25) QoL groups according to the baseline total PDQ-39 mean scores.

All analyses were performed using SAS version 9.4 software package (Statistical Analysis System, Cary, NC).

## Results

### Study selection and characteristics

**Fig. 1:**
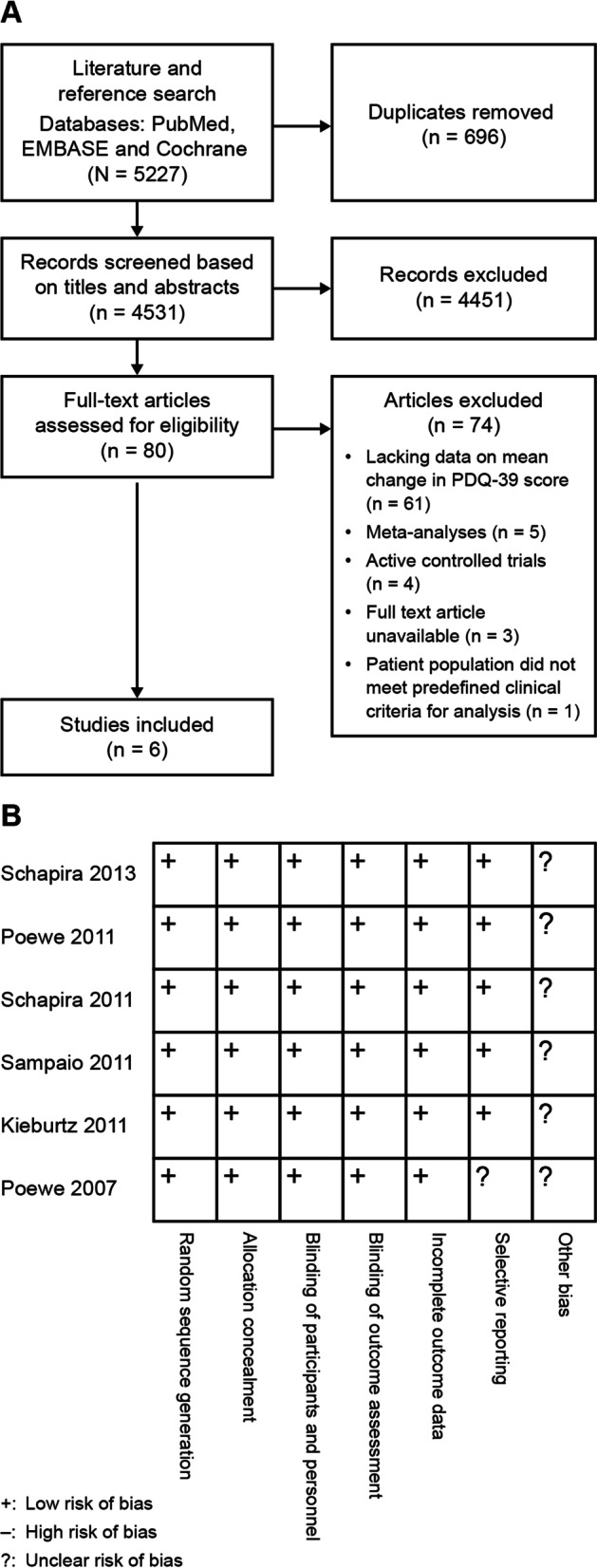
**A** Flow chart of study identification and inclusion for meta-analysis. **B** Quality assessments of included studies. PDQ-39, Parkinson’s Disease Questionnaire

A total of 5227 potential studies were identified following systematic literature searches of the three databases, which were filtered for duplicates (*n* = 696) and screened for eligibility (Fig. 1A). Full-text versions of the remaining 80 articles were further assessed, from which six studies were deemed eligible and included in the final meta-analysis [22–27].

The main characteristics of the six selected studies are summarised in Table 1. All the included studies were randomised, placebo-controlled, multicentred and parallel-group, and consisted of at least 2000 patients. Among these, four studies included patients with early PD and two involved those with advanced PD. Total treatment duration for each study ranged from 12 weeks to 9 months. Patients who were assigned to pramipexole received a dosage of 0.375–4.5 mg daily. The mean change in PDQ-39 between pramipexole and placebo was not available for one study [27] and two studies did not publish baseline PDQ total scores [22, 25]. Accordingly, the authors/study sponsors were contacted for the relevant PDQ scores although the final treatment differences in PDQ-39 between pramipexole and placebo for this analysis were derived from existing available treatment difference data that were published.

**Table 1:** Main characteristics of the included studies in the meta-analysis

Study	Country	Total N^a^	Mean age^b^ (SD)	Disease stage	Treatment duration	Treatment arms + background levodopa use		Follow-up duration
						**Treatment arms (n)**	**Levodopa**	
Schapira, 2013 [27]	Austria, Finland, France, Germany, Italy, Japan, Spain, Sweden, UK, USA	411	62.5 (10.0)	Early PD	9 months^c^	PBO (214)/PPX 1.5 mg daily (221)	None	65 weeks
Poewe, 2011 [22]	Argentina, Austria, Czech Republic, Finland, Germany, Hungary, India, Japan, Malaysia, Russia, Slovakia, Taiwan, Ukraine, USA	466	61.2 (9.7)	Early PD	33 weeks	PBO (103)/PPX ER (223) or IR (213), 0.375–4.5 mg daily	None (< 3 months of levodopa, discontinued before study)	26 weeks maintenance
Schapira, 2011 [23]	Austria, Czech Republic, Hungary, India, Italy, Philippines, Poland, Russia, Slovakia, South Korea, Spain, Sweden, Ukraine, UK	478	61.5 (9.9)	Advanced PD	18 weeks^d^	PBO (178)/PPX ER (164) or IR (175), 0.375–4.5 mg daily	Stable, with motor fluctuations	1 week
Sampaio, 2011 [26]	N/A	309	61.6 (10.3)	Early PD	24 weeks^e^	PBO (119)/pardoprunox 6 mg (112), 12 mg (115) and 12–42 mg (111) daily/PPX 1.5–4.5 mg daily (115)	None (discontinued > 3 months before baseline)	1 week
Kieburtz, 2011 [24]	USA	311	62.8 (10.2)	Early PD	12 weeks	PBO (77)/PPX 0.5 mg bid (81), 0.75 mg bid (73), 0.5 mg tid (80)	None	None
Poewe, 2007 [25]	Europe, South Africa, Australia, New Zealand	506	64.2 (9.6)	Advanced PD	16 weeks	PBO (100)/rotigotine 4–16 mg daily (201)/PPX 0.375–4.5 daily (200)	Stable, with motor fluctuations	4 weeks

^a^Total N is based on the number of available PDQ-39 data at the timepoint of interest according to the original publication

^b^Calculated from total mean age using the individual ages provided for separate treatment groups

^c^In this randomised delayed-start trial, patients were randomly assigned to receive PPX or placebo for 9 months. After 9 months, the placebo group received PPX and followed-up until 15 months. For this analysis, only the first 9-month data were included, which compared PPX to placebo [27]

^d^PDQ-39 data were only available at 18 weeks; the study follow-up periods include both 18 weeks and 33 weeks[23]

^e^The original publication included two randomised, controlled, double-blind studies with different follow-up periods (24 and 31 weeks). Follow-up data for PDQ-39 were only available for 24 weeks [26] bid, twice daily; ER, extended release; IR, immediate release; PBO, placebo; PD, Parkinson’s disease; PDQ-39, Parkinson’s Disease Questionnaire; PPX, pramipexole; tid, three times daily

### Risk of bias assessment

Figure 1B displays the risk of bias assessment for all six selected studies, which includes assessment of selection, performance, detection, attrition and reporting, as well as other potential sources of bias. The general methodological quality of the six studies was rated as acceptable, with adequate sequence generation, allocation concealment and double-blinded design. Only one study [25] was considered to carry a potential risk for selective reporting since missing data were not adequately addressed.

### Meta-analysis of the effect of pramipexole on QoL

In all six selected trials, PDQ-39 was reported as a secondary or other outcome; none reported PDQ-39 as a primary outcome. The mean change in PDQ-39 total score from baseline to last available value was extracted from all of the included studies, except Schapira et al. [27], which only published the median change in PDQ-39 total score (Table 2).

**Table 2:** Overview of PDQ-39 total scores for pramipexole and placebo

	**Baseline, mean (SD/IQR)**		**Change from baseline, baseline**–**follow-up (SE/95% CI)**		**Mean difference between treatment groups, PPX vs PBO (SE)**
	**PPX**	**PBO**	**PPX**	**PBO**	
Schapira, 2013^a^ [27]	12.5 (10.1)	12.2 (9.9)	–0.1 (7.3)	2.5 (9.0)	–2.60 (0.74)
Poewe, 2011^b,c^ [22]	ER: 30.8 (21.5) IR: 29.8 (20.8)	28.6 (22.8)	ER: –3.8 (–5.9 to –1.8) IR: –6.5 (–8.6 to –4.5)	–1.5 (–4.4 to 1.5)	–3.68 (2.19)
Schapira, 2011^c^ [23]	ER: 53.1 (27.6) IR: 53.3 (26.9)	51.1 (28.3)	ER: –9.1 (1.9) IR: –13.1 (1.8)	–6.2 (1.8)	–5.0 (3.12)
Sampaio, 2011 [26]	18.9 (13.8)	17.4 (14.9)	–0.9 (0.9)	0.1 (0.9)	–1.0 (1.18)
Kieburtz, 2011^c,d^ [24]	0.5 mg bid: 12.2 (8.9) 0.75 mg bid: 14.4 (9.1) 0.5 mg tid: 12.0 (8.3)	12.8 (11.5)	NA	NA	–1.72 (1.15)
Poewe, 2007^b^ [25]	32.9 (14.02)	34.8 (13.91)	–5.1 (10.1)	–1.3 (9.4)	–3.80 (1.18)

^a^Mean data obtained from clinical trial report (only median data reported in original publication)

^b^Baseline data for 2011 Poewe study was obtained from clinical trial report and for 2007 Poewe study from the study sponsor (UCB Biosciences)

^c^Mean differences between treatment groups are pooled (ER/IR and multiformulation studies)

^d^Change from baseline and mean difference between treatment groups data were not available in the original publication. Treatment effect was used for the analyses in this study. bid, twice daily; CI, confidence interval; ER, extended release; IQR, interquartile range; IR, immediate release; NA, not available; PBO, placebo; PD, Parkinson’s disease; PDQ-39, Parkinson’s Disease Questionnaire; PPX, pramipexole; SD, standard deviation; SE, standard error; tid, three times daily

**Fig. 2:**
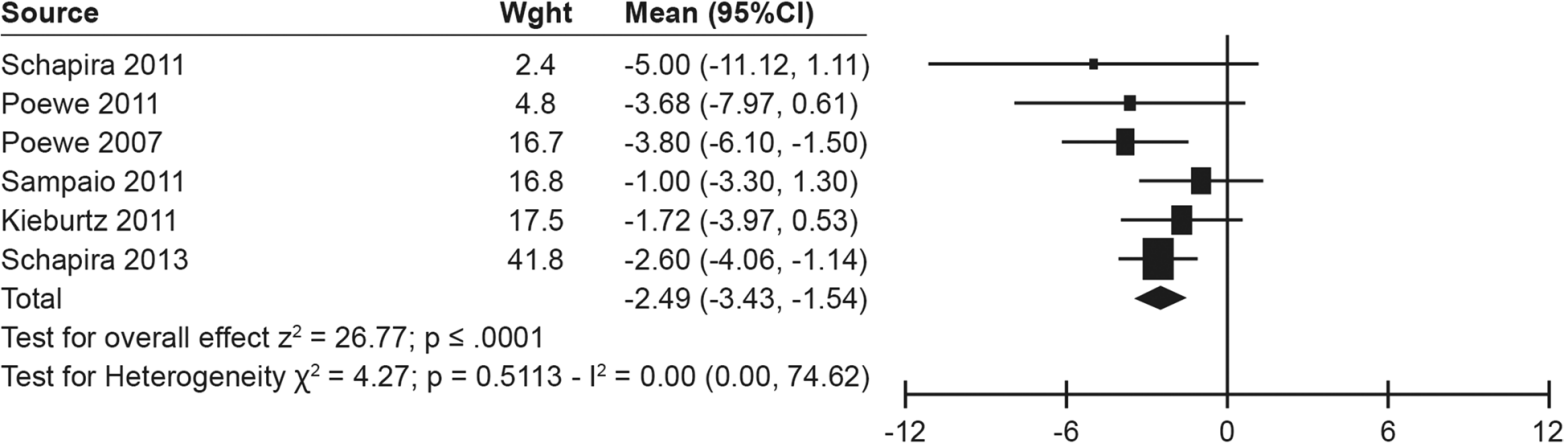
Effect of pramipexole versus placebo on PDQ-39 total score. CI, confidence interval; PDQ-39, Parkinson’s Disease Questionnaire

A significant mean change from baseline in the PDQ-39 total score of –2.49 (95% CI, –3.43 to –1.54; *p* < 0.0001) was observed with pramipexole compared with placebo (Fig. 2).

Sensitivity analyses excluded studies that may have included selective reporting bias [24] and the median values were reported instead of the mean [27]. Excluding these studies did not alter the main findings on the effect of pramipexole.

### Exploratory subgroup analyses on QoL

Further subgroup analyses were conducted to explore the effects of pramipexole on QoL according to treatment dose, baseline disease stage and baseline PDQ-39 total score.

**Fig. 3:**
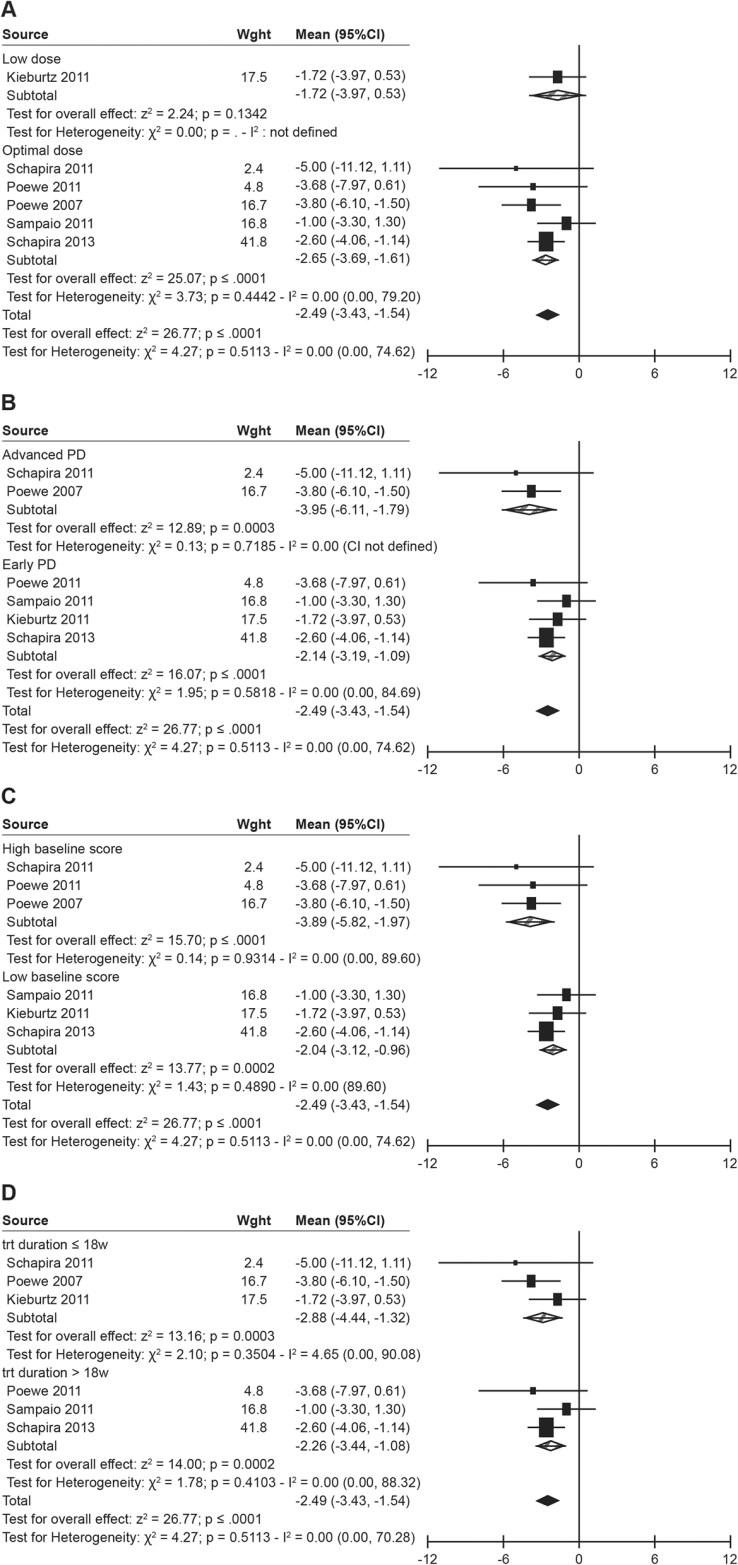
Effect of pramipexole versus placebo on PDQ-39 total score by predefined subgroups (treatment dose, baseline disease stage, baseline QoL level, treatment duration). **A** Effect of pramipexole versus placebo on PDQ-39 total score by treatment dose. **B** Effect of pramipexole versus placebo on PDQ-39 total score by baseline disease stage. **C** Effect of pramipexole versus placebo on PDQ-39 total score by QoL level at baseline. **D** Effect of pramipexole versus placebo on PDQ-39 total score by treatment duration

Pramipexole improved QoL compared with placebo where recommended optimal doses (≥ 80% on 1.5 mg) were prescribed (mean difference of –2.65; 95% CI, –3.69 to –1.61; *p* < 0.0001) in five studies [22, 23, 25–27] (Fig. 3A). No difference was observed for the low-dose group (–1.72; 95% CI, –3.97 to 0.53, *p* = 0.1342) [24]. In studies with early PD (mean difference of –2.14; 95% CI, –3.19 to –1.09; *p* < 0.0001) [22–27] and advanced PD (–3.95; 95% CI, –6.11 to –1.79; *p* < 0.001) [23, 25], pramipexole was shown to produce significant improvements in QoL compared with placebo (Fig. 3B). Regardless of whether baseline PDQ-39 scores were higher (≥ 25 points, worse QoL) [22, 23, 25] or lower (< 25 points, better QoL) [24, 26, 27], both of these subgroups reported greater improvements with pramipexole than with placebo (mean difference of –3.89; 95% CI, –5.82 to –1.97; *p* < 0.001 versus –2.04; –3.12 to –0.96; *p* < 0.001) (Fig. 3C). Considering the range of treatment duration in the included studies (12 weeks to 9 months), a post-hoc analysis to explore mean change in the PDQ-39 total score according to treatment duration found a consistent trend for the effect on QoL with pramipexole regardless of whether the studies had a shorter (≤ 18 weeks; mean difference of –2.88; 95% CI, –4.44 to –1.32; *p* < 0.001) or longer (> 18 weeks; –2.26; 95% CI, –3.44 to –1.08; *p* < 0.001) treatment duration (Fig. 3D).

## Discussion

This is the first comprehensive systematic review and meta-analysis to assess the effect of pramipexole on QoL in patients with PD. Our analysis pooled PDQ-39 total scores from six eligible pramipexole randomised controlled trials (RCTs) in patients with PD and demonstrated a greater mean change from baseline with pramipexole. In addition, a trend toward improvement in QoL with pramipexole was observed in patients who received optimal dosing (1.5 mg), regardless of their disease severity (advanced versus early) or baseline QoL levels.

Dopamine agonists can be administered either as monotherapy or adjunctive therapy for managing PD symptoms and also for reducing the risk of levodopa-related motor fluctuations and dyskinesia [8]. An earlier review that investigated the effect of pharmacotherapy for PD on QoL included three double-blind controlled studies of pramipexole that reported inconsistent effects of pramipexole versus the control on QoL. This was possibly due to the different types of control (levodopa or placebo) or QoL measures utilised in the reviewed studies (e.g., specific: PDQ-39, Parkinson’s Disease Quality of Life scale [PDQUALIF]; generic: EuroQol five-dimensional health-related quality of life measure [EQ-5D], Functional Status Questionnaire [FSQ], Medical Outcomes Study – 36-item Short Form survey [SF-36]) [12]. Nonetheless, a greater improvement in QoL with pramipexole (monotherapy or add-on) had been observed in early PD [12]. This was further investigated in a systematic review that included eight pramipexole clinical trials with QoL as a study outcome using a variety of QoL measures [9]. This systematic review provided some evidence that pramipexole can improve patients’ QoL when administered as an adjunct therapy; however, the magnitude of change could not be calculated based on the available data [9]. A subsequent narrative review evaluated the effect of a wider range of pharmacological treatments for PD on QoL, measured by PDQ-39 or PDQ-8 questionnaires [5]. The efficacy of pramipexole on QoL was demonstrated in two Level I studies of pramipexole vs other dopamine agonist or monoamine oxidase-B inhibitors and placebo; however, differences remained for other Level III studies [5]. Recent randomised studies have further suggested a beneficial effect on QoL with pramipexole, including a randomised trial comparing pramipexole/rasagiline combination therapy with different pramipexole doses [28] and a controlled trial with pramipexole as an adjunct therapy in PD inpatients; however, in the latter study, details of the randomisation, such as treatment dose and length of hospitalisation were unclear [29]. Since these previous reviews, our current search now consists of high quality eligible RCTs to investigate and quantify the effect of pramipexole alone versus placebo in patients with stable PD [22–27].

A comprehensive review by the Movement Disorder Society Task Force assessed a variety of PD-specific scales for QoL and found that PDQ-39 was the most widely used measure in PD research, with sufficient psychometric properties and validated in different populations [30]. The PDQ-39 correlates with motor disability and is impacted negatively by the total number of non-motor symptoms [31], and it has also been used to track longitudinal change in QoL in PD [32]. PDQ-39 was also used in the previous systematic review [9], and in consideration of its established and validated utility as a PD-specific QoL assessment tool, PDQ-39 was selected for this review. Briefly, scores for each dimension in the PDQ-39 are expressed as a summed item score ranging from 0 to 100, with a lower overall score reflecting better QoL [33]. Given the different baseline PDQ-39 scores for each study, this meta-analysis was performed on the change from baseline scores. The authors of the PDQ-39 also determined minimal clinically important difference (MCID) thresholds for the PDQ-39 total score and individual dimension scores based on responses to a survey of 728 patients with PD [34]. According to this study, a minimum of 1.6 points was considered to be the MCID threshold to indicate a clinically meaningful change in the PDQ-39 total score [34]. MCIDs are developed by benchmarking measurements or scores against subjective assessments reported by patients, and have been found to carry less bias than calculated effect sizes [35]. Using PDQ-39 as single disease-specific measure for QoL, our current analysis shows that pramipexole improves QoL in patients with PD compared with placebo, with a significant mean difference from baseline of –2.49 (95% CI, –3.43 to –1.54), which includes the MCID of –1.6.

Many factors related to QoL have been identified in patients with PD [36, 37]. We wanted to explore whether the beneficial effects of pramipexole on QoL remain in various subgroups of patients, such as patients at early/advanced disease stage or with higher/lower baseline QoL, as well as any effects of pramipexole dosing. The current analysis included four studies of patients with early PD and two studies of patients with advanced PD as defined at study inclusion. Our analysis revealed that treatment with pramipexole significantly improved QoL in patients with PD regardless of disease stage. Baseline QoL status has been shown to be associated with the level of decline in QoL over time in previous PD research [38, 39]. The current analysis demonstrated that QoL improved with pramipexole regardless of whether patients had better or worse QoL status at baseline. The labelling of pramipexole in the USA, UK and China [10, 11, 20] indicates that the treatment dose should be gradually titrated to achieve an optimal dose of 1.5–4.5 mg per day based on efficacy and tolerability. Our analysis found significant improvement in QoL among those who received the optimal maintenance dose of pramipexole (≥ 1.5 mg). Pramipexole 1.5 mg was determined as a critical dose since patients receiving ≥ 1.5 mg per day showed greater improvements in motor function and fewer adverse events than patients who received < 1.5 mg per day [21]. However, a daily dose lower than 1.5 mg has been reported in a previous clinical trial in China [40] and this remains a concern. The critical dose of 1.5 mg should be recognised and applied in clinical practice to ensure that the best treatment outcomes can be achieved for patients. Overall, our findings suggest that the benefits of pramipexole in patients with PD were generally consistent across groups with different baseline factors.

Despite the predominantly motor manifestations of PD, non-motor symptoms have been documented across the spectrum and disease stage of PD [36, 41, 42], including depression [36, 43] and sleep disturbance [39, 44]; these are identified as significant predictors of QoL scores. Studies have demonstrated a greater longitudinal influence of non-motor symptoms on QoL scores compared with motor symptoms [41, 42]. Pramipexole is established as a safe and effective treatment for PD across all disease stages [22–24, 45]. Among the dopamine agonists, pramipexole has a unique antidepressant effect and has demonstrated improvements in sleep disturbance independent of motor symptom improvement in PD [46–48]. Our analysis found significant improvements with pramipexole in both early and advanced PD subgroups. This suggests that pramipexole provides clinically meaningful improvements in QoL in patients with PD, which is important for long-term care and subjective overall well-being, in addition to disease symptom control, for this chronic incurable disease. Further investigations are needed to understand the effect of pramipexole on QoL through different motor and non-motor factors, as well as the change in contributing factors over the course of the disease.

This systematic review and meta-analysis applied rigorous and reproducible methods, and can be generalised to a broader profile of patients with PD. Nonetheless, a number of caveats should be considered when interpreting these results. The current results are based on six eligible trials, which may limit the overall power, although the total number of patients included was more than 2000. A fixed-effects model was applied to derive a weighted average of treatment effects estimated from individual studies, since heterogeneity was not detected for the included studies [17]. Due to the PDQ-39 mean difference from the 2013 study by Schapira et al. having the smallest variability (smaller SE and narrower CI) compared with the other studies included, the model assigned the greatest weight to the mean difference in this study for the meta-analysis calculation [27]. PDQ-39 as a self-reported measure of QoL outcome could have been affected by patients’ subjectivity [3] and does not capture environmental changes (e.g., clinical picture of disease, social status, social and living conditions, the number and intensity of social contacts) [5] that may have been experienced by a patient during the study. The PDQ-39 data for this analysis were not primary outcomes and were pooled from various RCTs with different study durations and designs and thus may have been influenced by other factors. For example, in the 2013 study by Schapira et al., patients in the delayed treatment arm (placebo) received the study drug (pramipexole) after 9 months; therefore, the treatment assignment is no longer true [27]. Additionally, in an earlier study by Schapira et al. [23], the PDQ data were not available at 33 weeks. We therefore performed a sensitivity analysis (Fig. 3) using 18 weeks as a cut-off (a standard pramipexole treatment follow-up duration [10]) and found a consistent effect on QoL with pramipexole. Finally, only studies published in English were considered and included in this analysis.

## Conclusion

This meta-analysis of pooled data from six pramipexole RCTs evaluating the change in PDQ-39 scores from baseline between pramipexole and placebo in patients with PD provides new evidence for a QoL benefit with pramipexole in PD. These results could be confirmed in larger pramipexole RCTs that investigate QoL as a major efficacy outcome of interest.

## Supplementary Information

**Additional file 1: Supplementary Figure S1.** Effect of pramipexole versus placebo on PDQ-39 total score by treatment dose. **Supplementary Figure S2.** Effect of pramipexole versus placebo on PDQ-39 total score by baseline disease stage. **Supplementary Figure S3.** Effect of pramipexole versus placebo on PDQ-39 total score by QoL level at baseline. **Supplementary Figure S4.** Effect of pramipexole versus placebo on PDQ-39 total score by treatment duration.

## Data Availability

Data used in this meta-analysis are available from the source studies as listed in the references section.

## Abbreviations

CI: Confidence interval
MCID: Minimal clinically important difference
PD: Parkinson’s disease
PDQ-39: Parkinson's Disease Questionnaire
QoL: Quality of life
RCT: Randomised controlled trial
SE: Standard error
