# Supplementary material for: Efficacy of pramipexole on quality of life in patients with Parkinson’s disease: a systematic review and meta-analysis
Source: BMC Neurol. 2022 Aug 25;22:320. doi: 10.1186/s12883-022-02830-y (PMC9404654; doi:10.1186/s12883-022-02830-y)
Supplement: Supplementary file 1 — Additional file 1: Supplementary Figure S1. Effect of pramipexole versus placebo on PDQ-39 total score by treatment dose. Supplementary Figure S2. Effect of pramipexole versus placebo on PDQ-39 total score by baseline disease stage. Supplementary Figure S3. Effect of pramipexole versus placebo on PDQ-39 total score by QoL level at baseline. Supplementary Figure S4. Effect of pramipexole versus placebo on PDQ-39 total score by treatment duration. [file 12883_2022_2830_MOESM1_ESM.docx]

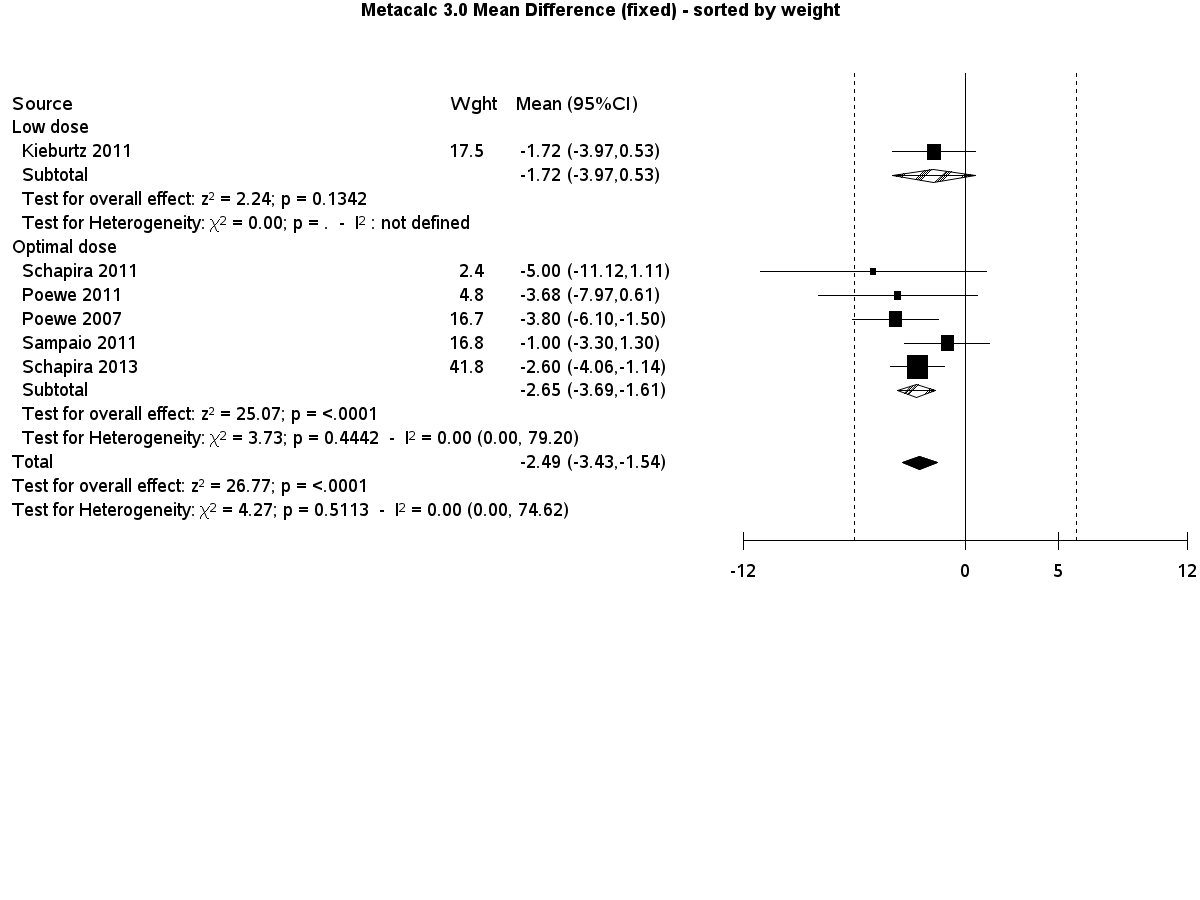


**Supplementary Figure S1. Effect of pramipexole versus placebo on PDQ-39 total score by treatment dose**


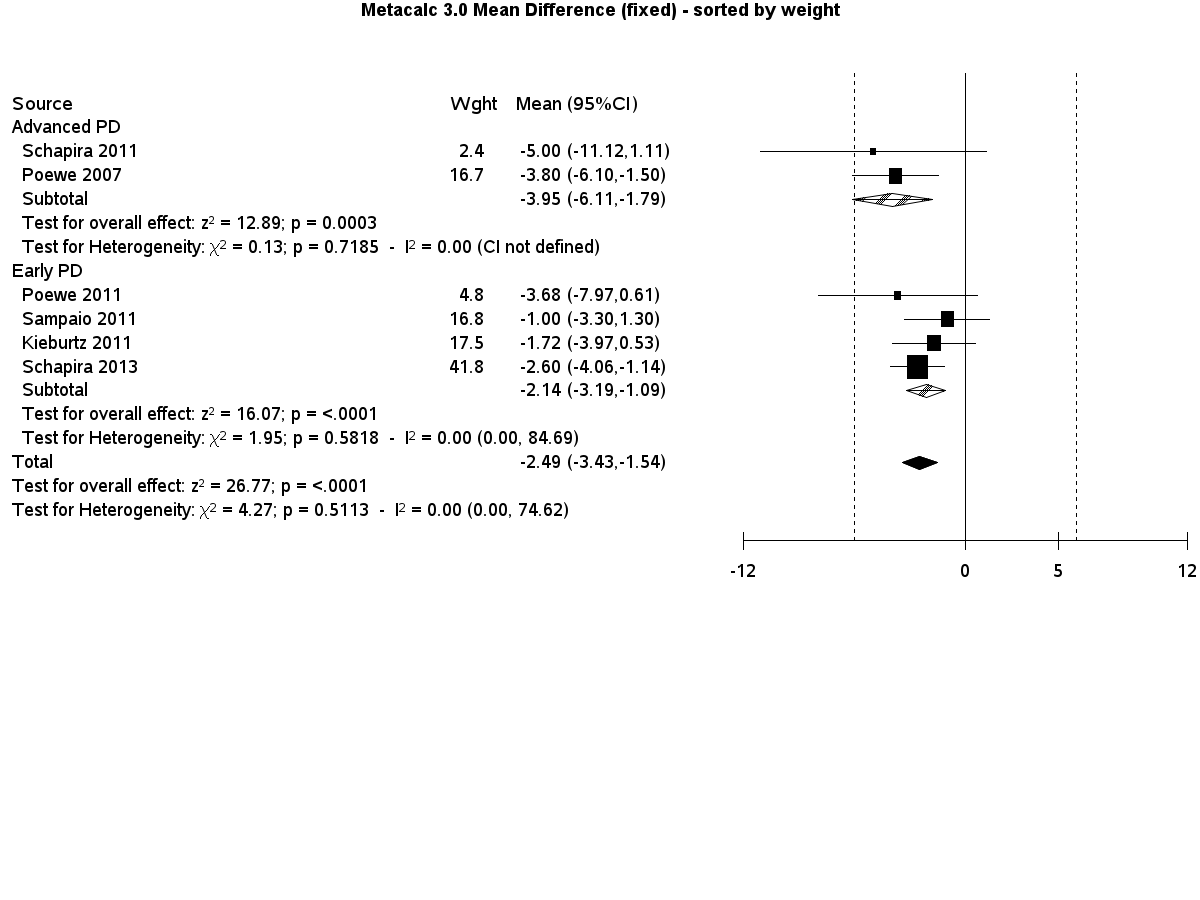


**Supplementary Figure S2. Effect of pramipexole versus placebo on PDQ-39 total score by baseline disease stage**


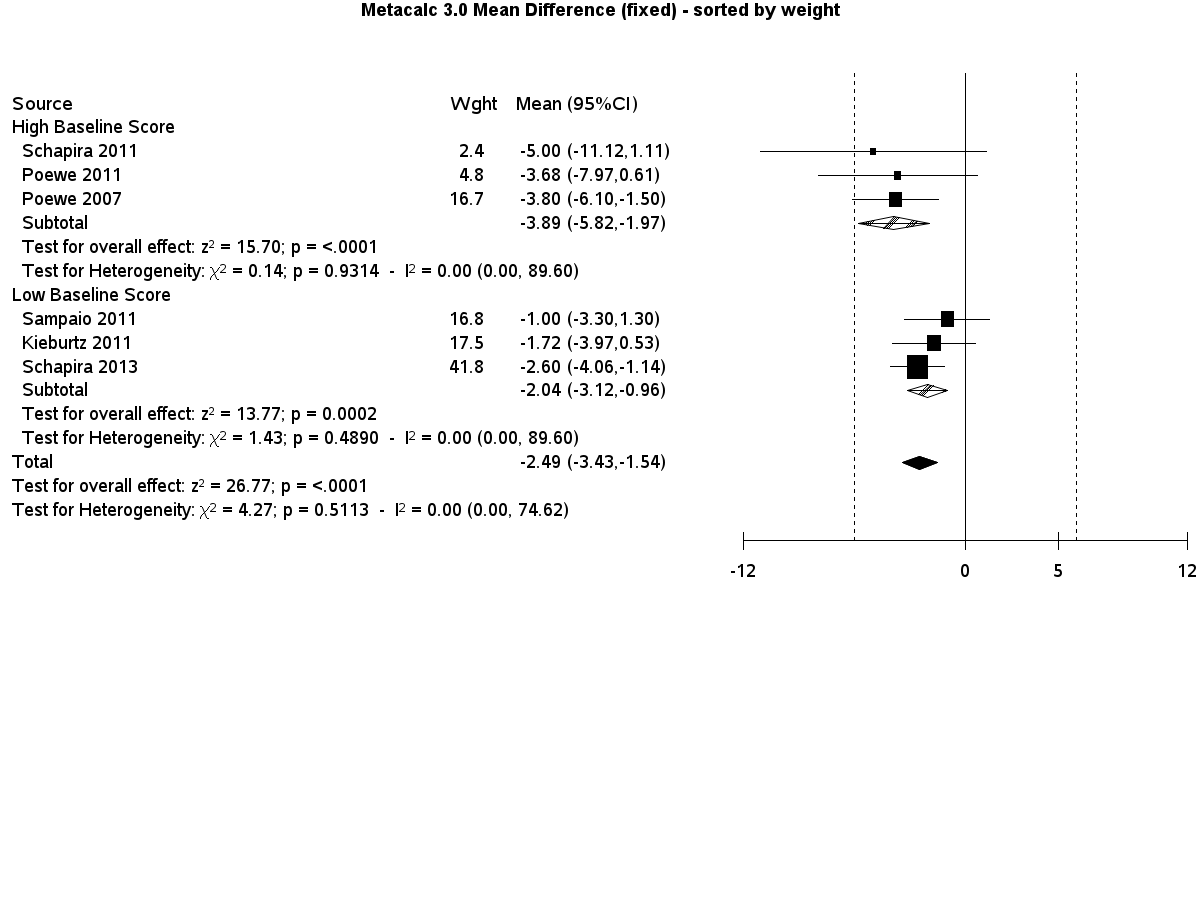


**Supplementary Figure S3. Effect of pramipexole versus placebo on PDQ-39 total score by QoL level at baseline**


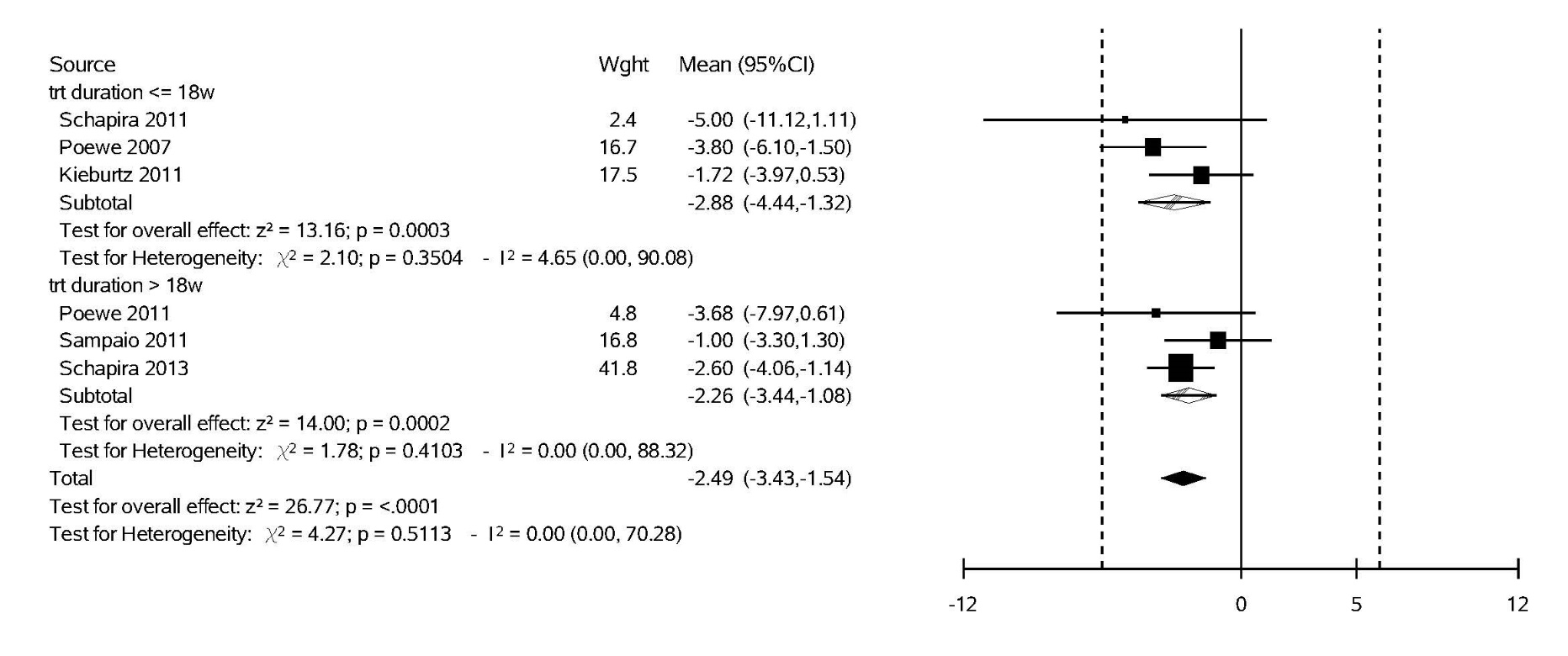
**Supplementary Figure S4. Effect of pramipexole versus placebo on PDQ-39 total score by treatment duration**
